# A Functional Analysis on the Interspecies Interaction between Mouse LFA-1 and Human Intercellular Adhesion Molecule-1 at the Cell Level

**DOI:** 10.3389/fimmu.2017.01817

**Published:** 2017-12-21

**Authors:** David Núñez, Laura Comas, Pilar M. Lanuza, Diego Sánchez-Martinez, Marta Pérez-Hernández, Elena Catalán, María Pilar Domingo, Adrián Velázquez-Campoy, Julián Pardo, Eva M. Gálvez

**Affiliations:** ^1^Immune Effector Cells Group, Aragón Health Research Institute (IIS Aragón), Biomedical Research Centre of Aragón (CIBA), Zaragoza, Spain; ^2^Department of Biochemistry and Molecular and Cell Biology, Fac. Ciencias, University of Zaragoza, Zaragoza, Spain; ^3^Instituto de Carboquímica ICB-CSIC, Zaragoza, Spain; ^4^Institute of Biocomputation and Physics of Complex Systems (BIFI), Unidad Asociada IQFR-CSIC-BIFI, Universidad de Zaragoza, Zaragoza, Spain; ^5^Aragón I + D Foundation (ARAID), Government of Aragon, Zaragoza, Spain; ^6^Nanoscience Institute of Aragon (INA), University of Zaragoza, Zaragoza, Spain; ^7^Department of Microbiology, Preventive Medicine and Public Health, University of Zaragoza, Zaragoza, Spain

**Keywords:** intercellular adhesion molecule-1, LFA-1, interspecies cross-reactivity, lymphocyte adhesion, integrins

## Abstract

The interaction between intercellular adhesion molecules (ICAM) and the integrin leukocyte function-associated antigen-1 (LFA-1) is crucial for the regulation of several physiological and pathophysiological processes like cell-mediated elimination of tumor or virus infected cells, cancer metastasis, or inflammatory and autoimmune processes. Using purified proteins it was reported a species restriction for the interaction of ICAM-1 and LFA-1, being mouse ICAM-1 able to interact with human LFA-1 but not human ICAM-1 with mouse LFA-1. However, *in vivo* results employing tumor cells transfected with human ICAM-1 suggest that functionally mouse LFA-1 can recognize human ICAM-1. In order to clarify the interspecies cross-reactivity of the ICAM-1/LFA-1 interaction, we have performed functional studies analyzing the ability of human soluble ICAM-1 and human/mouse LFA-1 derived peptides to inhibit cell aggregation and adhesion as well as cell-mediated cytotoxicity in both mouse and human systems. In parallel, the affinity of the interaction between mouse LFA-1-derived peptides and human ICAM-1 was determined by calorimetry assays. According to the results obtained, it seems that human ICAM-1 is able to interact with mouse LFA-1 on intact cells, which should be taking into account when using humanized mice and xenograft models for the study of immune-related processes.

Adhesion molecules are glycoproteins expressed on cell surfaces, where they mediate the contact between cell–cell, cell–extracellular matrix, and cell–pathogen. They are essential for physiological and pathological processes like embryonic development, wound healing, maintenance of tissue architecture, regulation of immune cell responses, migration of inflammatory cells into inflamed tissues, autoimmune diseases, and tumor metastasis (1, 2). Among the many molecules that regulate these processes, the interaction between the members of the intercellular adhesion molecule (ICAM) family and the integrin leukocyte function-associated antigen-1 (LFA-1, also known as CD11a/CD18) is especially relevant, ICAM-1 being the most extensively studied and best characterized member of ICAMs.

Intercellular adhesion molecule-1 is a type I transmembrane cell-surface protein, belonging to the immunoglobulin superfamily, with a molecular weight of 80–120 kDa depending on the level of glycosylation. The extracellular portion of ICAM-1 consists of 453 mainly hydrophobic amino acids, which form five immunoglobulin (Ig)-like domains. The extracellular region is attached to a single hydrophobic transmembrane region and a short cytoplasmic tail (3). ICAM-1 is the receptor for different human viruses like rhinovirus (4) and coxsackievirus A21 as well as for the malarial parasite *Plasmodium falciparum* (5) as well as different members of the integrin family like Mac-1 (6) and LFA-1 (7–10). Among them, the interaction with LFA-1 is the most critical step that mediates immune cell migration, activation, and target cell recognition.

Intercellular adhesion molecule-1 is mainly expressed as a dimer on the cell surface and dimerization appears to enhance binding to LFA-1 (11). Nevertheless, each individual ICAM-1 monomer is fully competent to bind LFA-1 and dimerization seems to be dispensable to form a complete LFA-1-binding site (12). Although ICAM-1 is usually anchored to the membrane, a soluble ICAM-1 molecule (sICAM-1) has been identified in serum. sICAM-1 is presented in serum from healthy humans at concentrations between 100 and 450 ng/ml (13) and increased levels of sICAM-1 have been found in serum from patients with cardiovascular and inflammatory diseases as well as during cancer metastasis (14, 15).

LFA-1 is a heterodimeric glycoprotein comprising a αL (CD11a, 180 kDa) and β_2_ (CD18, 95 kDa) subunits that are non-covalently linked. Both domains have a complex structure that includes large extracellular domains, single-pass transmembrane segments, and short intracellular tails (16). The α subunit of LFA-1 (αL) contains an N-terminal stretch of 200 amino acids, the inserted (I) or A domain, that is crucial for the ligand-binding specificity (17). A metal ion-dependent adhesion site (MIDAS) is located in the upper face of the I-domain (18). ICAM-1 interacts with LFA-1 through the binding of its first Ig-domain (D_1_) with the MIDAS within the I-(inserted) domain at the top of the N-terminus of αL subunit of LFA-1 (19). The ICAM-1/LFA-1 interaction is facilitated by magnesium and manganese divalent cations, assorted with five amino acids of MIDAS in LFA-1 and glutamate in domain 1 of ICAM-1 (20).

Interaction of LFA-1 in lymphocytes with ICAM-1 in target cells critically regulates all steps involved in the immune response including homing of lymphocytes, monocytes, and granulocytes during the inflammatory responses, Ag presentation, T helper and B lymphocyte responses, and T and natural killer (NK) cell-mediated killing (21, 22). In addition, it has been involved in several pathologies like metastasis of cancer cells or cardiovascular and autoimmune diseases.

Humanized mice are widely used as *in vivo* models to study the molecular basis of immune-related disorders as well as the efficacy of potential drugs (23). In these mice, the native gene of interest is replaced by its human homolog or the immune system is eliminated and reconstituted with its human counterpart. In addition, these mice can be used to enhance the engraftment of human cancer cells allowing *in vivo* studies of cancer progression and treatment. Here, a major obstacle to analyze human cell function, either healthy or transformed, and disease is the lack of species cross-reactivity of many growth factors, cytokines, or ligands required for development, survival, and function of the human grafted tissue/cells. Most of the cell adhesion molecules involved in leukocyte function are conserved across species. However, some of them like CD2 differ in tissue distribution and ligands (24, 25). LFA-I and ICAM-1 are well conserved across species including tissue distribution, ligands, and function (26–28). However, using purified cell-free models, it has been described that mouse ICAM-1 binds human LFA-1, but human ICAM-1 does not bind mouse LFA-1 (29). In contrast, other groups have shown that *in vivo* control of cancer development by mouse immune system is enhanced by expression of human ICAM-1 (30–32). Later, it was reported that murine LAK cells kill more efficiently mouse tumor cells transfected with human ICAM-1 than parental non-transfected cells (33). These results suggest a functional binding of human ICAM-1 to mouse LFA-1.

As indicated, homing and effector function of immune cells as well as cancer metastasis are critically regulated by LFA-1/ICAM-1 interaction and, thus, clarifying the species cross-reactivity of these molecules is crucial to support the utility of humanized mice in immune-related pathologies and cancer and to understand the results thereof. Here, we provide the functional experimental evidence showing that human ICAM-1 is able to interact with mouse LFA-1 during immune cell adhesion and cell cytotoxicity.

## Experimental Procedures

### ICAM-1 Chimeras and LFA-1-Derived Peptides

The synthetic first two domains of ICAM-1 alone (D_1_D_2_) or linked with human IgG1 Fc region (D_1_D_2_Fc) were expressed in *Escherichia coli* and purified by single step column refolding as described (34). We have previously shown that the affinity of the interaction of human D_1_D_2_Fc with human LFA-1 is similar to a chimera formed by the five extracellular domains of ICAM-1, D_1_D_5_Fc (35). Mouse and human Fc chimeras containing the five extracellular domains of ICAM-1 (D_1_D_5_Fc) were purchased in R&D systems.

Human and mouse LFA-1-derived peptides (human: CD11a_237–261_; mouse: CD11a_235–259_) were purchased from GenScript (Piscataway, USA) without any modification. The sequences of CD11a_237–261_ and CD11a_235–259_ are ITDGEATDSGNIDAAKDIIRYIIGI and ITDGEASDKGNISAAHDITRYIIGI, respectively, and have been previously characterized in basis of its ability to block ICAM-1/LFA-1 interaction (36).

### Mouse Strains

Mice (C57BL/6J) between 5 and 7 weeks of age were used. These mice were maintained and bred in pathogen-free conditions in the animal facility of the Center for Agriculture Research and Technology (CITA) of the Government of Aragón. Mice were maintained on a 12 h light–dark cycle. Each cage contained aspen woodchip bedding (Capsumlab) and nesting material (shredded paper), and food (Harlan Laboratories) and tap water were provided *ad libitum*.

All procedures were approved by the Ethic Committee for Animal Experiments from CITA. The care and use of animals were performed accordingly with the Spanish Policy for Animal Protection RD53/2013, which meets the European Union Directive 2010/63 on the protection of animals used for experimental and other scientific purposes.

### Isothermal Titration Calorimetry (ITC)

The ability of the human chimera D_1_D_2_Fc to interact with a peptide derived from its natural ligand LFA-1 in mouse was analyzed by ITC on a VP-ITC calorimeter (MicroCal) at 25°C as indicated previously (34). Briefly, 2.2 ml of a 12-µM solution of D_1_D_2_Fc and 0.5 ml of a 150-µM solution of peptide were prepared in PBS and degassed. Each assay consisted of a series of 28 injections of peptide solution of 10 µl each (with a 4-µl first injection) at 400 s intervals under constant stirring (459 rpm). The thermal power required to keep the cell at a constant temperature is measured, so that it provides the heat associated with each ligand injection after integrating the signal over time. The thermodynamic parameters of protein–peptide interactions (affinity, enthalpy, and entropy changes) as well as the stoichiometry were estimated by using nonlinear regression analysis. Data were analyzed using the software developed and implemented in Origin 7.0 (OriginLab).

### Analysis of ICAM-1 and LFA-1 Interaction by Flow Cytometry

The ability of human D_1_D_2_Fc and mouse and human D_1_D_5_Fc to recognize LFA-1 on mouse cells was analyzed by flow cytometry in the mouse thymic lymphoma cell line EL4. EL4 cells were incubated with different concentrations of human D_1_D_2_Fc, mouse and human D_1_D_5_Fc and human IgG1 control at 4°C for 45 min, washed twice with FACS buffer (PBS, 5% FCS, 0.1% NaN_3_), and then incubated with PE-conjugated goat anti-human IgG (Fcγ fragment specific; Jackson ImmunoResearch). After a washing step with FACS buffer, cells were analyzed by FACS using a FACS Calibur with CellQuest Pro software (BD). As control cells were incubated with an antibody against mouse CD11a (LFA-1) before adding human D_1_D_2_Fc.

### Human and Mouse Macrophage Differentiation

Monocytes were isolated from human peripheral blood mononuclear cells (PBMCs), obtained from healthy donors (Blood and Tissue Bank of Aragon; approved by the CEICA, number: C.I.PI11/006) by density gradient centrifugation using Ficoll-Paque (GE healthcare). PBMCs were resuspended at a concentration of 2.5 × 10^6^ cells/ml in RPMI medium supplemented with 1% heat-inactivated human AB serum (Sigma). Cells were cultured in a Petri dish for 2 h at 37°C 5% CO_2_. Then cells were carefully washed twice with PBS, and RPMI medium supplemented with 10% FBS, 2 mM l-glutamine, and antibiotics (penicillin 100 U/ml, streptomycin 100 µg/ml) (complete RPMI medium) was added and cells were further incubated for 2 h at 37°C 5% CO_2_. Cells were washed again with PBS and complete RPMI medium supplemented with GM-CSF (Invitrogen) at a concentration of 0.1 ng/ml was added. Cell culture was maintained for 6 days until monocytes are differentiated into macrophages. Macrophage differentiation was confirmed by microscopic evaluation under light microscope and by immunophenotypical characterization using the following antibodies from BD Pharmingen: HLA-DR-PE, CD14-FITC, ICAM-1-FITC, and LFA-1-APC.

Mouse macrophages were differentiated from mouse bone marrow as previously described (37). Briefly, bone marrow cells isolated from mouse femur were cultured in DMEM 10% FBS, 5% horse serum, and 30% supernatant of L929 cell culture as a source of M-CSF at a density of 10^6^/cm^2^ for 7–8 days. Macrophage differentiation was analyzed in the same way as human macrophages but using CD11b-APC and CD11c-PE (BD) antibodies.

### Cell Adhesion Assays

Freshly isolated monocytes or differentiated macrophages were washed twice with PBS and incubated with trypsin (PAN Biotech) for 30 min at 37°C 5% CO_2_. After that, cells were scraped gently from the plate and resuspended at a final concentration of 10^6^ cells/ml in complete RPMI medium. Subsequently, 10^5^ macrophages and 5 × 10^5^ monocytes were added to each well and incubated with several concentrations of human D_1_D_2_, D_1_D_2_Fc, D_1_D_5_Fc, mouse D_1_D_5_Fc, control human IgG1 20 µg or lovastatin 100 µM at 37°C 5% CO_2_ overnight. Next day, every well was washed twice with PBS, complete RPMI medium was added and the number or adherent remaining cells was quantified by a MTT colorimetric test (38). In some experiments, plates previously coated with human and mouse D_1_D_5_Fc were used. To coat the plates 5 µg of D_1_D_5_Fc in 100 µl of PBS was incubated overnight at 4°C and subsequently plates were washed with PBS and blocked with 1% BSA in PBS for 2 h at room temperature.

### NK Cell-Mediated Cytotoxicity

The effect of human and mouse peptides derived from LFA-1 on ICAM-1/LFA-1 dependent cell–cell contact was analyzed by performing a cell cytotoxicity assay using human primary NK cells and the NK cell sensitive human leukemia K562. Primary activated human NK cells were generated by culturing PBMCs with mitomycin C inactivated R69 cells for 5 days and enriched by MACS using anti-CD56 antibodies as previously described (39). Cell cytotoxicity induced by NK cells on K562 was analyzed by flow cytometry as previously described (39). Briefly, K562 cells were preincubated with medium alone, in the presence of 250 µM of human and mouse peptides from LFA-1 or D_1_D_2_ (15 µg) for 45 min at 37°C. Then, NK cells were added at 3:1 effector:target cell ratio and incubated for 4 h at 37°C, 5% CO_2_. Subsequently, phosphatidylserine exposure and 7-AAD uptake were analyzed by FACS using the annexin-V/7-AAD kit from Immunostep. As a control, the cells were incubated alone with the inhibitors to evaluate their possible toxicity, which was not significant in any case.

### CD8^+^ T Cell-Mediated Cytotoxicity

To check whether the human chimeras of ICAM-1 and human and mouse peptides from LFA-1 were able to recognize their counterpart in mouse cells, a cytotoxicity assay with lymphocytic choriomeningitis virus (LCMV)-specific CD8^+^ T cells generated *in vivo* and EL4 cells as targets as previously described (40) was carried out. Mice were inoculated intraperitoneally (i.p.) with 10^5^ plaque-forming units LCMV–WE in 200 µl. At day 8 post-infection, mice were sacrificed by cervical dislocation, the spleens were removed, splenocytes were isolated, and CD8 cell fraction was enriched by MACS, using anti-CD8-MicroBeads (Miltenyi Biotec). Subsequently, CD8 cells were resuspended in RPMI medium 5% decomplemented FBS and preincubated in the presence of several concentrations of human D_1_D_2_, D_1_D_2_Fc, mouse D_1_D_5_Fc, 250 µM of human and mouse LFA-1 derived peptides, human IgG1 15 µg and lovastatin 100 µM for 45 min at 37°C 5% CO_2_. EL4 cells were previously incubated for 2 h at 37°C 5% CO_2_ with 1 µM of the immunodominant peptide of LCMV, KAVYNFATM (gp33, NeoMPS). Then, CD8 cells and EL4 target cells were incubated at ratio effector:target 10:1 in 96-well plate conical bottom for 4 h at 37°C 5% CO_2_. Subsequently, phosphatidylserine exposure and 7-AAD uptake were analyzed by FACS using the annexin-V/7-AAD kit from Immunostep. As a control, EL4 cells and CD8 lymphocytes were incubated in the absence of gp33. In addition, in both cases, the cells were incubated alone with the inhibitors to evaluate their possible toxicity, which was not significant in any case.

### B Cell Aggregation Assay

The ability of human and mouse peptides from LFA-1 and human ICAM-1 to inhibit cell homotypic aggregation was analyzed by using mouse B cells. To obtain these cells, mice were sacrificed by cervical dislocation, the spleens were removed, and splenocytes were isolated and resuspended in RPMI 10% FBS at a final concentration of 10^6^ cells/ml. Cells were treated with 10 µg/ml PHA and 1 µg/ml ionomycin to activate LFA-1 and incubated at 37°C 5% CO_2_ overnight in flat bottom 96-well plates (10^5^ cells) in the presence of peptides from LFA-1 and c-Myc (control) 250 µM, D_1_D_2_, D_1_D_2_Fc, and human IgG1 10 µg, EGTA 1 mM and lovastatin 100 µM. A qualitative aggregation assay was carried out as described (36, 41) with a slight modification. Briefly, positive control stimulated cell samples were arbitrarily assigned a clumping index of 10 and lovastatin samples an index of 1. Test samples were ranked from 1 to 10 based upon degree of clumping relative to stimulated and lovastatin-treated samples. Results were verified by a blind independent rating performed by a second observer.

### Statistic Analysis

Statistical analysis was performed using GraphPad Prism software (GraphPad, San Diego, CA, USA) by two-way analysis of variance, followed by Bonferroni’s posttest.

## Results and Discussion

### Interaction between Human ICAM-1 and Mouse Derived LFA-1 Peptides by ITC

In a previous work, we have shown that purified human recombinant ICAM-1 was able to interact with peptides derived from human LFA-1 (34). In order to test whether mouse LFA-1 contained sequences that are able to recognize human ICAM-1, we have now analyzed by ITC the interaction between human ICAM-1 and a peptide derived from mouse LFA-1. This peptide contains the sequence of mouse LFA-1 corresponding to the peptide of human LFA-1 that showed the highest affinity for human ICAM-1 (34).

**Figure 1:**
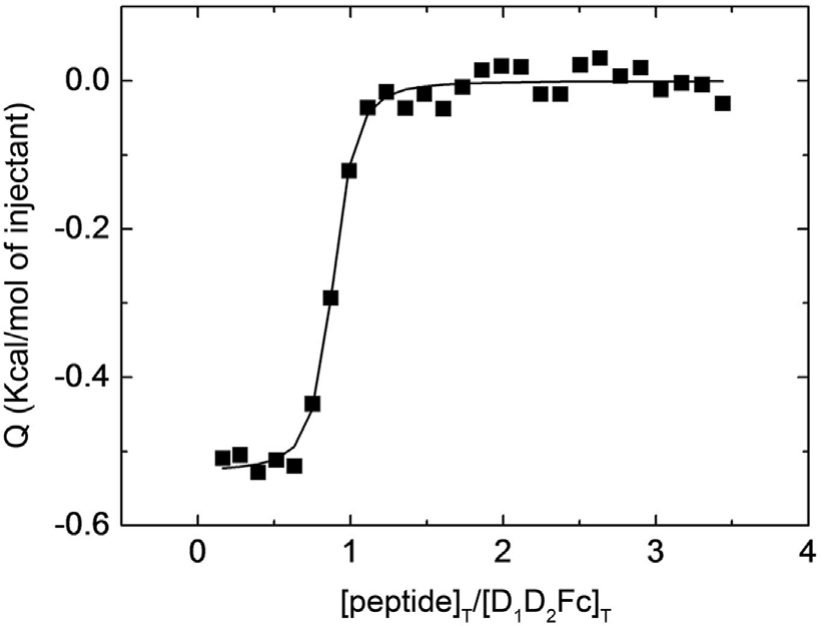
Interaction of LFA-1 peptide with D_1_D_2_Fc by isothermal titration calorimetry. Purified D_1_D_2_Fc (12 µM) was titrated with a peptide derived from the binding site of mouse LFA-1 (CD11a_235–259_; 150 µM). The assay was performed in PBS buffer at 25°C. The nonlinear regression analysis done as described in Section “Experimental Procedures” provided a dissociation constant of 70 nM.

As shown in Figure 1, the curvature of the binding isotherm implies a high affinity for D_1_D_2_Fc (*K*_D_ = 70 nM) similar to the one observed previously with a peptide derived from human LFA-1 (34). The complex formation was net exothermic as would be expected for a reaction in which specific intermolecular interactions are established. Besides, if there were no interaction, there would not be any signal. This value was obtained by fitting to the binding isotherm a 1:*n* interaction model, which assumes a single type of binding sites. This result indicates that mouse LFA-1 contains sequences that could interact with human ICAM-1 and suggests that mouse LFA-1 could potentially bind human ICAM-1.

### Binding of Human ICAM-1 with Cell-Associated Mouse LFA-1

**Figure 2:**
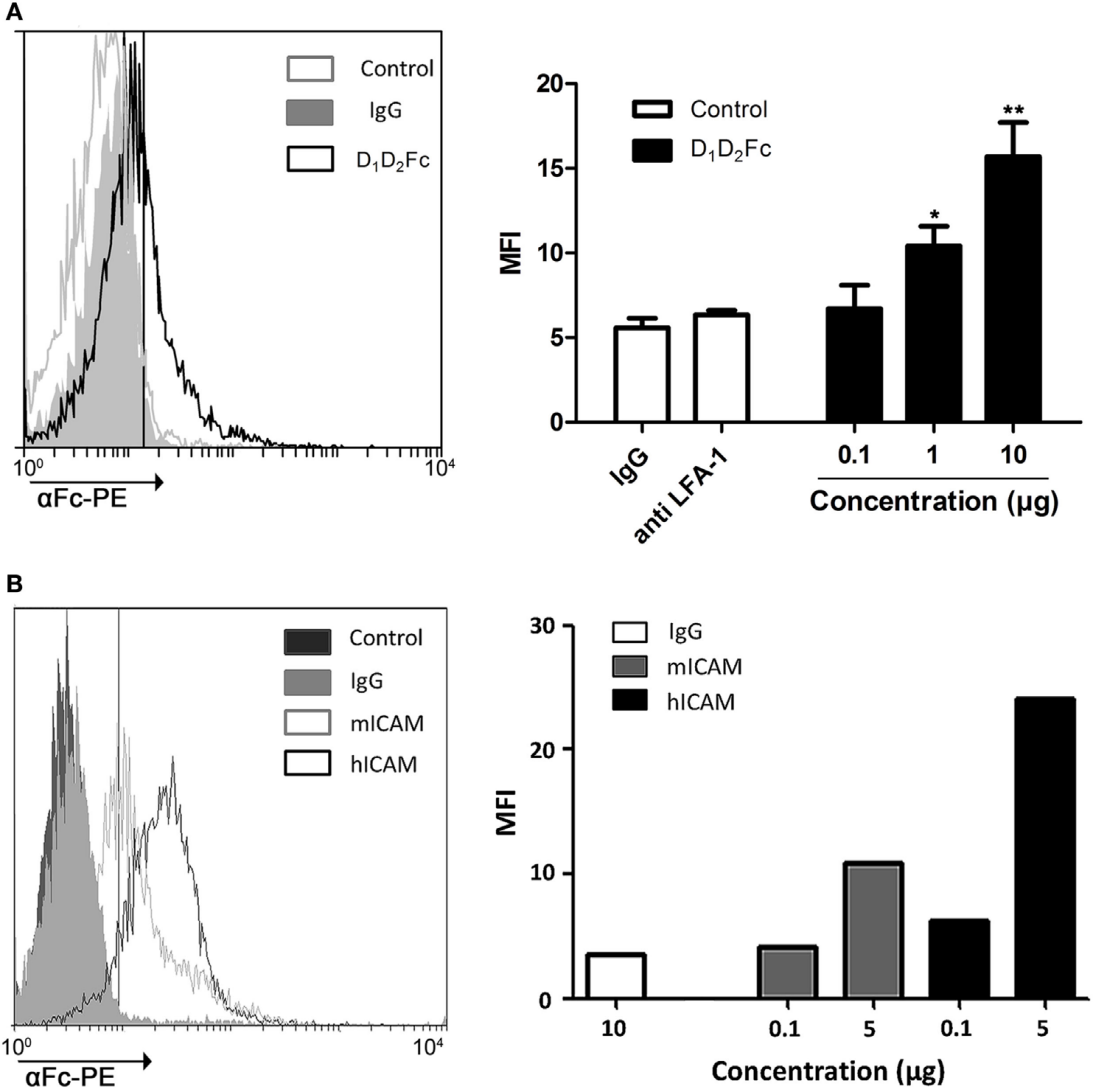
Analysis of D_1_D_2_Fc binding to LFA-1 in EL4 cells by flow cytometry. **(A)** Different amounts of D_1_D_2_Fc (0.1, 1, and 10 µg) or human IgG1 control (10 µg) were incubated with EL4 cells. After washing out non-bound proteins, cells were incubated with PE-conjugated goat anti-human IgG Fcγ Ab and analyzed by flow cytometry. As a control EL4 cells were pretreated with a mouse LFA-1 blocking antibody (anti LFA-1) before incubating with 10 µg of D_1_D_2_Fc. Histograms show a representative experiment. Values in the graph are represented as mean ± SEM of two independent experiments performed by duplicate. Statistical analysis was performed with two-way analysis of variance with Bonferroni’s posttest by comparing IgG with D_1_D_2_Fc. *p < 0.05; **p < 0.01. **(B)** Different amounts of mouse or human D_1_D_5_Fc (mICAM, hICAM; 0.1 and 5 µg) or human IgG1 control (10 µg) were incubated with EL4 cells. After washing out non-bound proteins, cells were incubated with PE-conjugated goat anti-human IgG Fcγ Ab and analyzed by flow cytometry. Histograms show a representative experiment.

Isothermal titration calorimetry experiments suggest that human ICAM-1 is able to recognize a specific sequence derived from αI-domain of the mouse integrin LFA-1 corresponding to the area of attachment to ICAM-1. However, although mouse LFA-1 derived short peptides have a high sequence homology in their counter-partner in human and human ICAM-1 is capable of recognizing them, these linear amino acid sequences do not completely mimic the interaction among proteins containing a three-dimensional spatial structure. Thus, to prove that human ICAM-1 is capable of interacting with mouse LFA-1, we have analyzed the ability of the purified protein to bind the native form of LFA-1 expressed on the plasma membrane of mouse lymphoid cells. To this aim, we tested the interaction of D_1_D_2_Fc and LFA-1 in the mouse T cell line EL4 which express high levels of LFA-1 (data not shown) by flow cytometry (Figure 2A).

As shown in Figure 2A, the percentage of positive cells increases as it increases the amount of ICAM-1 chimera added, indicating that human ICAM-1 is able to recognize and bind to mouse LFA-1 in compare with the negative control IgG. Incubation cells with a mouse antiCD11a (LFA-1) antibody blocked binding of the chimera.

To analyze the binding affinity of human ICAM-1 to mouse LFA-1, we compared the binding of mouse D_1_D_5_Fc and human D_1_D_5_Fc to EL4 cells. As shown in Figure 2B, both human and mouse ICAM-1 bound to EL4 cells, although in this case binding of human ICAM-1 was higher than binding of mouse ICAM-1.

To further evaluate and confirm that human ICAM-1 was able to interact with mouse LFA-1 in other cell types, we analyzed the ability of human ICAM-1 (D_1_D_2_Fc and D_1_D_2_) to block B cell aggregation by using a homotypic cell aggregation assay. Homotypic B cell aggregation is mostly due to ICAM-1/LFA-1 interaction and this assay is widely used to analyze the affinity of LFA-1 and/or ICAM-1 ligands (7).

**Figure 3:**
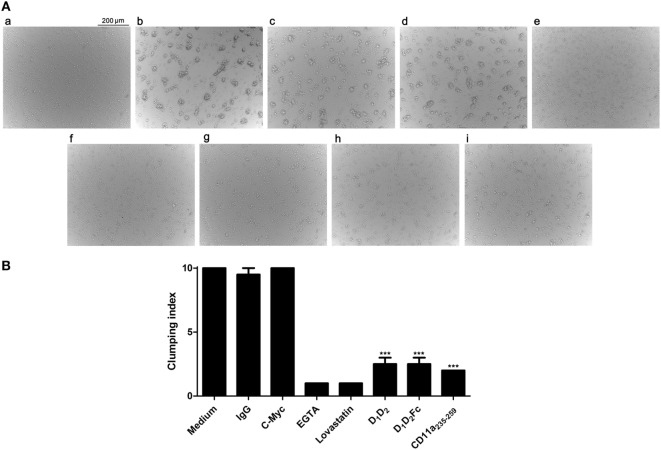
Effect of human (ICAM-1) on the aggregation of mouse spleen cells. **(A)** Splenocytes isolated from mice were non treated (a) or activated with PHA (b–i) as described in Section “Experimental Procedures” and further incubated overnight with (b) medium; (c) IgG1; (d) c-Myc peptide; (e) EGTA; (f) lovastatin; (g) D_1_D_2_; (h) D_1_D_2_Fc; (i) mouse LFA-1 peptide CD11a_235–259_. Images were taken with an optical microscope (original magnification, 10×). Representative images from two independent experiments with similar results are shown. **(B)** The graph represents the rate of agglutination depending on the inhibitory effect on ICAM-1/LFA-1 interaction. Cells stimulated with PHA and ionomycin, which served as a positive control, were arbitrarily assigned agglutination index 10, which represents a percentage of aggregation between 90 and 100%. Homotypic aggregation was classified from 1 to 10 depending on the degree of aggregation relative to cells stimulated. The result was verified by a second independent observer. Values are presented as mean ± SEM of two separate experiments performed by duplicate. Statistical analysis was performed using two-way analysis of variance with Bonferroni’s posttest comparing with c-Myc peptide control. *p < 0.05; ***p < 0.001.

Fresh mouse B cells were obtained from splenocytes and stimulated with PHA and ionomycin to induce homotypic adhesion. The images of cell aggregation assay are shown in Figure 3A, a–i. The results were quantified by establishing a clumping index that represents the degree of cell aggregation, which reflects the inhibition efficiency (Figure 3B). Cell aggregation was ranked by a clumping index from 1 to 10 based on the degree of aggregation in comparison with the stimulated cells (positive control, clumping index = 10).

As expected, non-stimulated fresh B cells did not aggregate in culture (Figure 3A, a). In contrast, cell aggregates were clearly visible in cultures after stimulation (Figure 3A, b). As controls, the cation chelator EGTA and the drug lovastatin (Figure 3A), which have been described to specifically block ICAM-1/LFA-1 interaction without affecting other adhesion molecules such a Mac-1 or VLA-4 (42, 43), were able to inhibit completely the aggregation, while human IgG1 and a peptide derived from c-Myc (Figure 3A, c,d) did not present any effect. Besides, incubation cells with a mouse antiCD11a (LFA-1) antibody blocked aggregation (data not shown). Human ICAM-1 (Figure 3A, g,h) significantly inhibited adhesion at concentration of 10 µg confirming that, as in the case of T cells, human ICAM-1 interacts with mouse LFA-1 in B cells. The mouse LFA-1 derived peptide (Figure 3A, i) completely blocked B cell aggregation confirming that this process was dependent on ICAM-1/LFA-1 interaction.

Our data using intact mouse cell expressing LFA-1 contrast with a previous work indicating that human ICAM-1 did not bind to mouse LFA-1 (29). Two explanations could account for these *a priori* contradictory results: (i) the previous work was performed using purified proteins and was not confirmed in intact cells or (ii) human ICAM-1 is binding to other integrins in EL4 or B cells like Mac-1. However, this is discarded since Mac-1 would bind to domain 3 in ICAM-1 and the proteins used here only consist of the first two domains. Thus, our results suggest that the interaction between human ICAM-1 and mouse LFA-1 in intact cells is different from that observed using purified proteins. Our data are in line with previous works suggesting that mouse LFA-1 interact with target cells expressing human ICAM-1 (30–32).

### Blocking of Cell-Mediated Cytotoxicity

As above mentioned, it has been previously reported that elimination of target cells by mouse cytotoxic lymphocytes is enhanced by overexpression of human ICAM suggesting that mouse LFA-1 is interacting with human ICAM-1 (33). Upon recognition of target cells, cytolytic leukocytes, NK, and mouse cytotoxic CD8^+^ T (Tc) cells, adhere to target cells before antigen recognition takes place (44), and these cells might utilize regulated changes in adhesion to control immune recognition events (45). The main adhesion molecule that plays a key role in the interaction with target cells is ICAM-1. The interaction of ICAM-1, expressed on tumor target cells, with LFA-1, expressed on the membrane of NK cells and Tc cell, modulates the formation and signaling of the immunological synapse and the subsequent elimination of the target cell (46).

**Figure 4:**
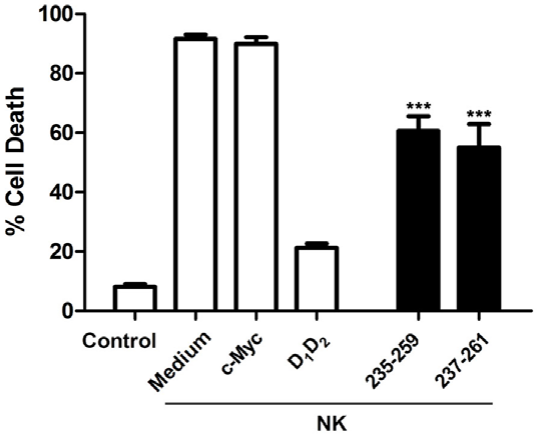
Inhibition of natural killer (NK) cell-mediated cytotoxicity by human intercellular adhesion molecule-1. Human NK cells isolated from healthy donors were activated and enriched by MACS as described in Section “Experimental Procedures” and labeled with the fluorescent probe CFSE. Subsequently they were incubated with K562 cells at effector:target (e:t) ratio 3:1 for 4 h in the presence or absence of human D_1_D_2_ or mouse LFA-1 peptide CD11a_235–259_ (250 µM), human LFA-1 peptide CD11a_237–261_ (250 µM), and c-Myc peptide as control. Cell death was analyzed by measuring translocation of PS by flow cytometry in the negative population for CFSE as described in experimental procedures. Values are presented as mean ± SEM of two independent experiments performed by duplicate. Statistical analysis was performed using two-way analysis of variance with Bonferroni’s posttest comparing with c-Myc control. *p < 0.05; ***p < 0.001.

First of all, we analyzed if the mouse LFA-1 derived peptide was able to block human NK cells mediated cytotoxicity on human K562 leukemic cells. As shown in Figure 4, this peptide was able to significantly inhibit cell death induced by human NK cells on the human leukemia cell line K562. This finding indicates that the peptide from mouse LFA-1 binds to human ICAM-1 in K562 cells preventing its interaction with LFA-1 on NK cells. As expected, a peptide derived from human LFA-1 as well as purified human ICAM-1 (D_1_D_2_) blocked NK cell-mediated cell death confirming the participation of ICAM-1/LFA-1 interaction in this process. Here, we only analyzed the effect of D_1_D_2_ to avoid a potential antibody dependent cell cytotoxicity effect when employing D_1_D_2_Fc quimera.

**Figure 5:**
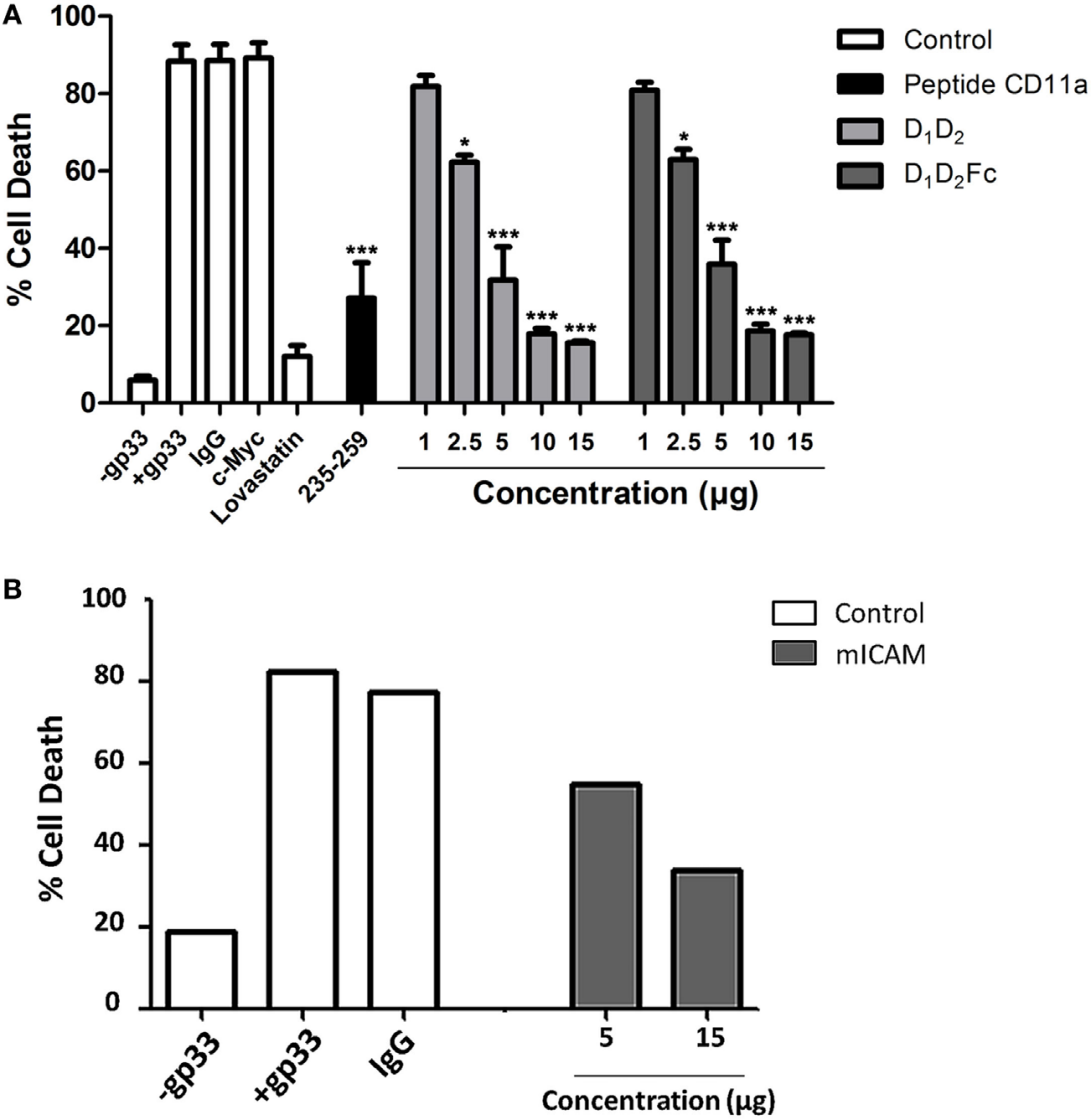
Inhibition of cytotoxic CD8^+^ T cell-mediated cytotoxicity. **(A)** CD8^+^ cells were isolated by MACS from mice previously infected with lymphocytic choriomeningitis virus as described in Section “Experimental Procedures” and labeled with the fluorescent probe CTG. Subsequently they were incubated with EL4 cells previously labeled with gp33 viral peptide at a e:t ratio 10:1 for 4 h in the presence or absence of different concentrations of human D_1_D_2_ and D_1_D_2_Fc, mouse LFA-1 peptide CD11a_235–259_, C-Myc 250 µM, IgG 15 µg, and lovastatin 100 µM. Cell death was analyzed by measuring translocation of PS by flow cytometry in the negative population CTG as described in experimental procedures. Values are presented as mean ± SEM of three independent experiments performed by duplicate. Statistical analysis was performed using two-way analysis of variance with Bonferroni’s posttest compared to the control IgG1 and c-Myc peptide for protein and the peptide, respectively. *p < 0.05; ***p < 0.001. **(B)** The same experiment as in **(A)** was performed but employing mouse intercellular adhesion molecule-1 (mICAM, D_1_D_5_Fc).

Next, we employed mouse Tc cells to analyze the effect of human purified ICAM-1 on its cytotoxic function. We have tested if human purified ICAM-1 was able to block cell death induced by LCM virus-specific Tc cells on mouse EL4 cells that express high levels of ICAM-1 (data not shown). As shown in Figure 5A, incubation with increasing amounts of human ICAM-1 completely blocked cell death induced by mouse Tc cells on LCMV gp33 antigen pulsed EL4 cells indicating that human ICAM-1 is interacting with mouse Tc cell-associated LFA-1 and preventing its binding to ICAM-1 on EL4 target cells. The peptide from mouse LFA-1 was also able to inhibit cytotoxicity confirming the implication of ICAM-1/LFA-1 interaction in this process. As additional controls, lovastatin and mouse ICAM-1 (D_1_D_5_Fc) inhibited cell death induced by Tc cells confirming the importance of ICAM-1 and LFA-1 during cell death induced by Tc cells. Mouse D_1_D_5_Fc (B) and human D_1_D_2_ and D_1_D_2_Fc (A) similarly prevented cell death induced by Tc cells indicating a similar affinity of human and mouse ICAM-1 to mouse LFA-1 in this model. In both cases, human NK cells and mouse Tc cells, the effect of the peptides was less pronounced than that of ICAM-1, which maybe explained due to a reduced capacity to block the interaction between ICAM-1 and LFA-1 by a short peptide in contrast to full ICAM-1.

Our results help to explain the previous observations in which expression of human ICAM-1 in mouse cancer cells favored its elimination *in vitro* and *in vivo* mediated by mouse immune cells including NK cells (30–33). These results suggested that mouse LFA-1 interacts with human ICAM-1. Here, we have confirmed this suggestion and shown that human ICAM-1 efficiently binds to mouse LFA-1 also in cytotoxic cells, preventing its function during cell-mediated elimination of target cells.

### Blocking of Monocyte/Macrophage Cell Adhesion

The growth of adherent cells such as monocytes or macrophages requires signals not only from growth factor receptors but also from integrins (47). Integrins have two basic roles: to mediate adhesion and signaling (48, 49). Indeed, it has been described that LFA-1 is one of the most critical molecules involved in monocyte/macrophage adhesion (49). To further proof that human ICAM-1 is able to bind mouse LFA-1, we decided to analyze this binding in another type of leukocytes, the macrophages. Thus, we analyzed the ability of human ICAM-1 (D_1_D_2_ and D_1_D_2_Fc) to block adhesion of mouse macrophages to plastic surfaces. As control, human monocytes and macrophages were used.

**Figure 6:**
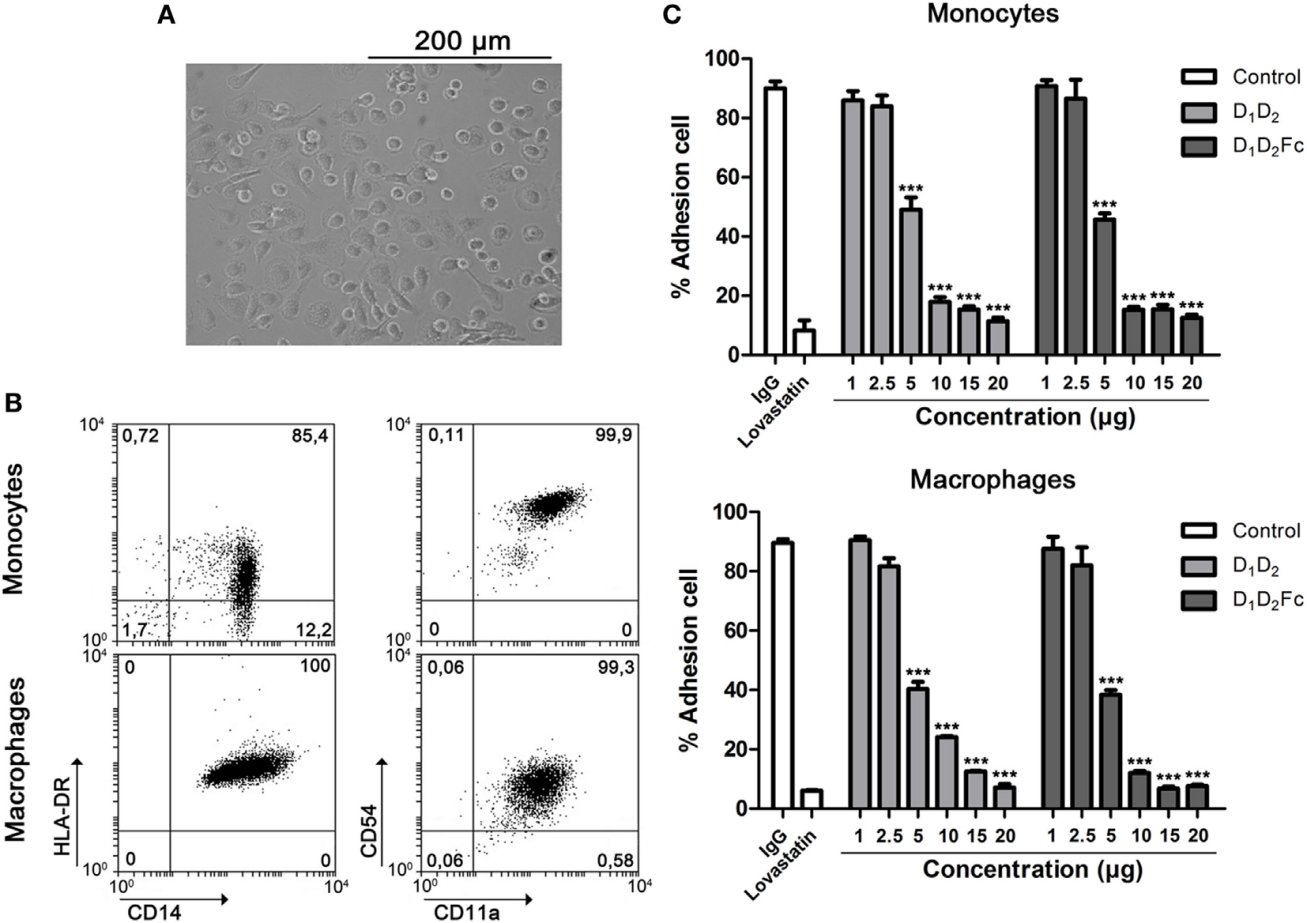
LFA-1 is involved in human macrophage adhesion. **(A)** Human monocytes were differentiated for 6 days in the presence of GM-CSF as indicated in Section “Experimental Procedures.” Representative images from cell cultures are shown (original magnification, 20×). **(B)** Macrophage phenotype was analyzed by flow cytometry testing the expression of HL-DR, CD14, CD54, and CD11a. A representative dot plot is shown. Numbers correspond to percentage of cells in each quadrant **(C)**. Monocytes and macrophages were incubated at different concentrations of human D_1_D_2_ and D_1_D_2_Fc, human IgG1 20 µg and lovastatin 100 µM at 37°C overnight. Subsequently, every well was washed twice with PBS to remove non-adherent cells and quantification of cell adhesion was carried out by MTT as described in Section “Experimental Procedures.” Values are presented as mean ± SEM of three independent experiments performed by duplicate. Statistical analysis was performed using two-way analysis of variance with Bonferroni’s posttest comparing with IgG1 control. ***p < 0.001.

As shown in Figure 6A, cells differentiated from human CD14^+^ monocytes to macrophages in the presence of GM-CSF had a classical adherent “fried egg” morphology, characteristic of classically activated M1 pro-inflammatory macrophages (50). Macrophage phenotype was confirmed by testing surface antigens by flow cytometry. Monocyte-derived macrophages expressed higher levels of HLA-DR and CD14 than undifferentiated monocytes (51). In addition, expression of CD54 (ICAM-1) was diminished in comparison with freshly isolated monocytes (Figure 6B). CD11a (LFA-1) expression did not change. As shown in Figure 6C, adhesion of human monocytes (Figure 6C, top) and macrophages (Figure 6C, bottom) was completely inhibited by human ICAM-1 confirming that macrophages adhere to plastic by LFA-1. As control, lovastatin was able to block completely cell adhesion demonstrating that inhibition of LFA-1 is sufficient to block macrophage adhesion. It should be noted here that other integrins like Mac-1 or VLA-4 are not inhibited by lovastatin (43).

In spite of the elevated expression of ICAM-1 in fresh monocytes in comparison with macrophages, it did not correlate with the adhesion abilities of both cells (data not shown). As indicated previously, since we have used an ICAM-1 form consisting of D_1_ and D_2_, we can disregard that ICAM-1 is preventing macrophage cell adhesion by blocking Mac-1 that is also known to be involved in macrophage cell adhesion (52, 53).

**Figure 7:**
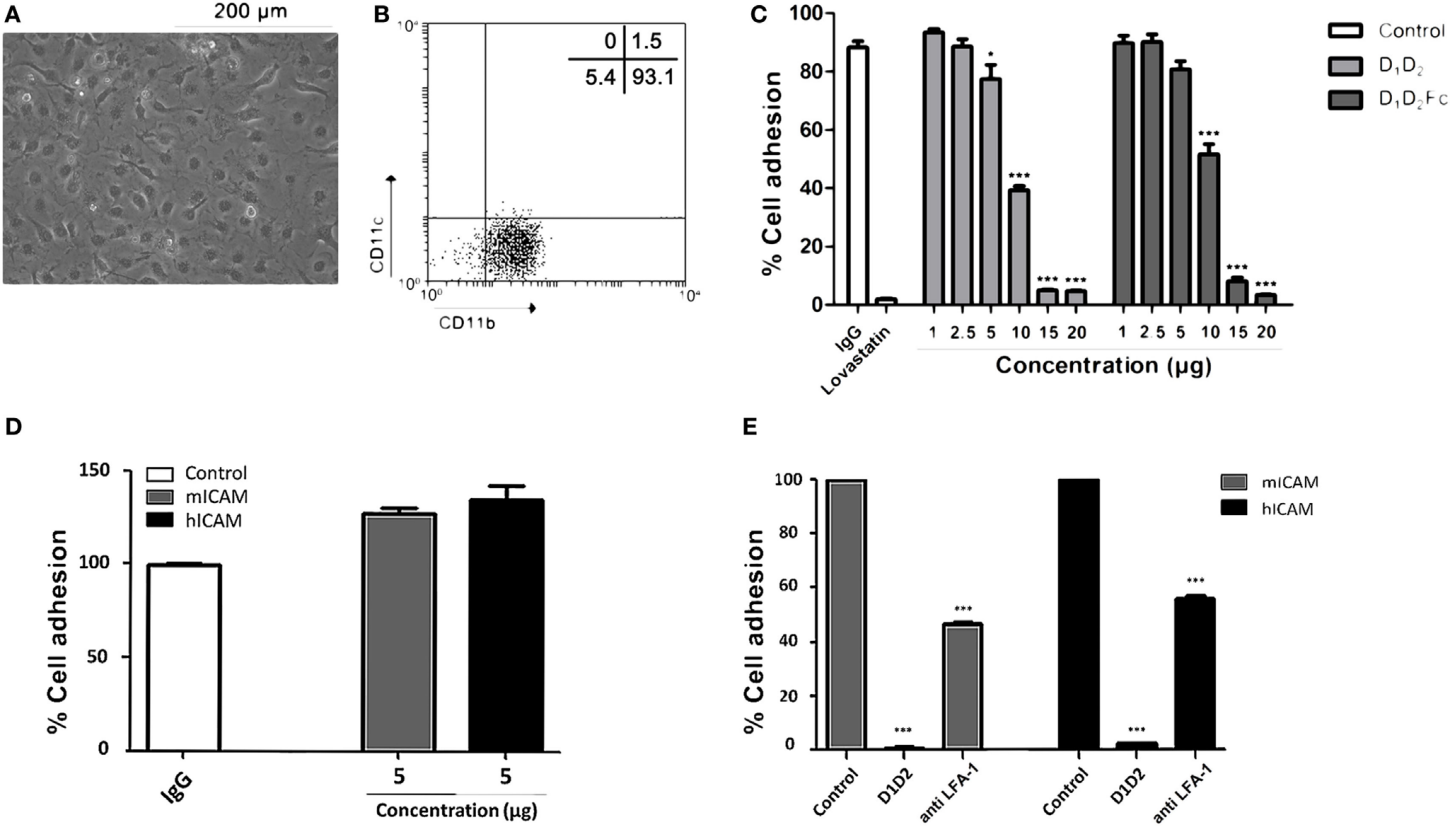
Human intercellular adhesion molecule (ICAM)-1 blocks LFA-1-mediated mouse macrophage cell adhesion. **(A)** Mouse macrophages were differentiated from bone marrow cells as described in Section “Experimental Procedures.” Representative images from cell cultures are shown (original magnification, 20×). **(B)** Macrophage phenotype was analyzed by flow cytometry testing the expression CD11b and CD11c. A representative dot plot is shown. Numbers correspond to percentage of cells in each quadrant. **(C)** Macrophages were incubated at different concentrations of human D_1_D_2_ and D_1_D_2_Fc, human IgG1 20 µg and lovastatin 100 µM at 37°C overnight. Subsequently, every well was washed twice with PBS to remove non-adherent cells and quantification of cell adhesion was carried out by MTT as described in Section “Experimental Procedures.” **(D,E)** The same experiment as in C was performed but using plates previously coated with 5 µg of mouse or human ICAM-1 (mICAM and hICAM; D_1_D_5_Fc) as indicated. **(D)** % of cell adhesion in wells coated with human IgG, mouse D_1_D_5_Fc (mICAM) or human D_1_D_5_Fc (hICAM). **(E)** Effect of human D1D2 and anti-mouse LFA-1 on macrophage cell adhesion to mouse D_1_D_5_Fc (mICAM) or human D_1_D_5_Fc (hICAM)-coated plates. Values are presented as mean ± SEM of two separate experiments performed by duplicate. Statistical analysis was performed using two-way analysis of variance with Bonferroni’s posttest comparing with IgG1 control. *p < 0.05; ***p < 0.001.

After confirming that human ICAM-1 inhibits adhesion of human macrophages by binding to LFA-1, the same test was carried out with mouse macrophages in order to support that human ICAM-1 is able to functionally bind to mouse LFA-1. Differentiation of mouse bone marrow cells to macrophages with M-CSF was morphologically (Figure 7A) and phenotypically (Figure 7B) confirmed. As shown in Figure 7B, these cells exhibited a characteristic phenotype of macrophages (CD11b^+^/CD11c^−^) (37). Again, D_1_D_2_ and D_1_D_2_Fc were able to inhibit the adhesion of mouse macrophages although less efficiently than in the case of human macrophages (Figure 7C). Lovastatin, an inhibitor of LFA-1, was able to completely inhibit the adhesion of these cells demonstrating that LFA-1 is the main molecule involved in macrophage adhesion also in the mouse system.

In this experimental model, uncoated plastic plates were used to analyze if human ICAM-1 was able to bind to mouse LFA-1 and, thus, interfere with macrophage attachment. However, in this experimental system, macrophages could adhere to plastic through different adhesion molecules. Thus, to really proof that human ICAM-1 was interfering with LFA-1-dependent mouse macrophage attachment, we repeated this experiment employing mouse macrophages and plates coated with mouse or human ICAM-1 (D_1_D_5_Fc). As shown in Figure 7D, mouse macrophages similarly adhered to plates independently whether they were coated with mouse or human ICAM-1. Moreover, pre-incubation of macrophages with either anti-LFA-1 Ab or human soluble ICAM-1 (D1D2) (Figure 7E) blocked macrophage adhesion in both mouse and human ICAM-1 coated plates, confirming that human ICAM-1 is also binding to mouse LFA-1 in macrophages.

It should be noted here that soluble human ICAM-1 (D1D2) completely blocked macrophage adhesion, indicating that the interaction of Mac-1 (CD11b) with the domain 3 of immobilized ICAM-1 is not enough to support macrophage adhesion. Indeed it has been previously found that LFA-1, and not Mac-1, is mainly involved in cell adhesion to ICAM-1 coated plates (54), which was related to the relatively weak affinity of Mac-1 to ICAM-1. In contrast, both LFA-1 and Mac-1 similarly mediated cell adhesion to ICAM-3-coated plates. The differences found between the effect of anti-LFA-1 Ab and human soluble ICAM-1 (D1D2) could be related to a different affinity to LFA-1 or to a different stoichiometry since blocking Ab was not fully titrated to find out an optimal concentration that completely blocks macrophage adhesion. Whatever the reason, these results clearly show that mouse macrophages bound to human ICAM-1-coated plates by a mechanism dependent on LFA-1 and, thus, human ICAM-1 is able to interact with mouse LFA-1 expressed on the membrane on macrophages.

It has been reported that human ICAM-1 shares a 53% structural homology with murine ICAM-1 (28). Concerning amino acid sequences, the first two domains of human ICAM-1 has around 70% identity or homology with mouse ICAM-1. Despite these comparisons, it has been shown that human ICAM-1 does not bind to mouse LFA-1 using *in vitro* cell-free systems (29). This finding contrast with other reports suggesting that functionally human ICAM-1 is able to interact with mouse LFA-1 during tumor immunosurveillance (30, 31, 33, 55). Although we have not measured direct interaction between human ICAM-1 and murine LFA-1 expressed both in different cells, our results show that human ICAM-1 is able to interact specifically with mouse LFA-1 using *in vitro* cell models and lovastatin as control of a specific interaction. This binding has also been confirmed at the functional level in a wide variety of cells expressing LFA-1 including transformed T cells and primary B, NK, and T cells as well as macrophages.

Although we do not have yet an explanation for the apparent contradictory findings, our results indicate that human ICAM-1 is able to interact with mouse LFA-1 at the cellular level. Besides, the results obtained are independent of the organism used to produce D_1_D_2_ because the domain 1, which is the binding site to LFA-1, does not require posttranslational modifications like glycosilation to bind LFA-1 (56, 57).

This finding should be taken into account when designing experiments and interpreting results using transgenic mice expressing human ICAM-1 or xenograft models in which host leukocytes expressing LFA-1 will bind to endogenously expressed human ICAM-1 transgene or to grafted human cells expressing ICAM-1.

## Ethics Statement

All procedures were approved by the Ethic Committee for Animal Experiments from CITA. The care and use of animals were performed accordingly with the Spanish Policy for Animal Protection RD53/2013, which meets the European Union Directive 2010/63 on the protection of animals used for experimental and other scientific purposes.

## Author Contributions

DN performed and designed experiments, analysed the data and wrote the first draft of the paper. LC, PML and DSM performed and designed experiments. MPH assisted in the generation and characterization of mouse macrophages. EC generated the LCMV-specific cytotoxic T lymphocytes. MPD established the conditions for ICAM-1 purification. AVC performed the ITC experiments and analysed the data. JP and EMG conceived the study and wrote the final version of the manuscript. All authors read and approved the final manuscript.

## Conflict of Interest Statement

The authors declare that the research was conducted in the absence of any commercial or financial relationships that could be construed as a potential conflict of interest. The reviewer ZF and handling editor declared their shared affiliation.
